# Diagnosis of keratoconus in a young male by electrophysiological test findings: A case report

**DOI:** 10.1097/MD.0000000000029351

**Published:** 2022-07-08

**Authors:** Weiming Yan, Yanjin Chen, Xiaohong Chen, Qian Ye, Yutong Wang, Chuan Jiang, Xiangrong Zheng, Yunpeng Wang, Meizhu Chen

**Affiliations:** a Department of Ophthalmology, The 900th Hospital of Joint Logistic Support Force, PLA (Clinical Medical College of Fujian Medical University, Dongfang Hospital Affiliated to Xiamen University), Fuzhou, China.

**Keywords:** case report, corneal topography, electroretingram, flash visual-evoked potential, keratoconus, pattern visual-evoked potential

## Abstract

**Rationale:**

The purpose of this report was to describe the diagnostic process of a case of keratoconus (KCN) after electrophysiological examination.

**Patient concerns:**

A 23-year-old male army officer presented with decreased visual acuity (VA) in the left eye for 5 months. Best-corrected VA was 20/20 in the right eye and 20/300 in the left eye. The cornea and lens were clear in both eyes with a normal anterior chamber. No specific abnormalities were found on fundus photography, optical coherence tomography, fundus fluorescein angiography (FFA), indocyanine green angiography (ICGA), or full-field electroretinography (ffERG) of both eyes. Pattern visual-evoked potentials (PVEP) detected a reduced amplitude and delayed peak time of the P100-wave in both eyes, which was more severe in the left eye. The amplitude and peak time of the P2-wave in flash VEP (FVEP) were comparable in both eyes and were within the normal ranges.

**Diagnosis:**

Corneal topography was performed, and KCN was diagnosed by the presence of an asymmetrical bowtie pattern in both eyes, which was worse in the left eye.

**Interventions:**

Transepithelial corneal collagen cross-linking was performed.

**Outcomes:**

The BCVA of both eyes remained stable after treatment at follow-up.

**Lessons:**

KCN should be suspected in cases of unimproved VA and significant irregular stigmatism, while no obvious lesions exist in other parts of the eyes. The evidence of lesion location by electrophysiological examinations could sometimes be of favor in diagnosing KCN.

## 1. Introduction

Keratoconus (KCN) is a common corneal ectasia characterized by thinning and protrusion of the cornea. It usually presents as a bilateral, asymmetric, and noninflammatory corneal disorder. KCN can lead to significant visual impairment, with the development of irregular astigmatism. Usually, visual acuity (VA) correction with glasses cannot be achieved. KCN usually starts at puberty, occurs in the second decade of life, progresses, and stabilizes in the third or fourth decade of life.^[1]^ Its prevalence and incidence vary widely among both sexes and ethnicities. The incidence is estimated to be between 50 and 230 per 100,000 people.^[2,3]^

Under a slit lamp, the conical protrusion sign of the cornea in KCN might not be easily observed in some cases.^[4]^ In these circumstances, KCN patients with unimproved VA can be undiagnosed or have delayed diagnosis. KCN was diagnosed unless the clinician came up with the idea of corneal tomography. Corneal tomography can assess the corneal surfaces and enable the detection of early or subclinical KCN.^[5]^

Electrophysiological examinations, which are especially popular in undiagnosed cases, have been used in clinics for centuries.^[6]^ Specifically, electroretinogram (ERG) induced by flash stimuli of series strength is a widely used test of retinal function.^[7]^ Visual-evoked potentials (VEP) with pattern-reversal checkboard (PVEP) or flash (FVEP) stimuli can provide an assessment of the functional integrity of the visual system, including the ocular media, retina, optic nerve, and visual cortex.^[8]^ The application of ERG and VEP could provide clues regarding the location of ocular lesions, which would aid in the diagnosis of ocular diseases in combination with other ophthalmic examinations.^[9,10]^

In this report, a case of KCN was finally diagnosed via corneal topography after the hint of the electrophysiological tests described. In addition, this report provides a review of the current literature regarding the application of electrophysiological examinations in patients with KCN.

## 2. Case report

A 23-year-old male army officer presented to our clinic complaining of decreased VA in his left eye over 5 months. He had no history of ocular diseases or ocular surgery. Moreover, he reported no ocular symptoms such as itching, redness, photophobia, or tearing. He and his family provided informed consent for the publication of the case. Besides, the report of our study was reviewed and approved by the Ethics Committee of the 900th Hospital of Joint Logistic Support Force, PLA.

**Figure 1.**
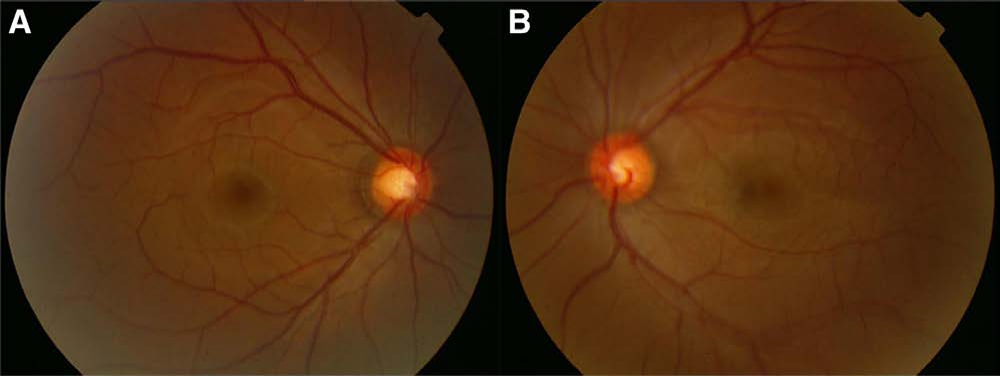
Fundus photograph of both eyes [(A) the right eye, OD; (B) the left eye, OS]. No obvious fundus abnormalities were present in both eyes.

Clinic evaluation showed that his best-corrected VA (BCVA) was 20/20 with −4.50 sph −0.75 cyl 40° in the right eye (OD). The BCVA was 20/300 in the left eye (OS), which could not be improved by correcting the refractive error (−19.00 sph −4.25 cyl 45°). The intraocular pressures detected by the noncontact tonometer (NTC) were 6 mm Hg OD and 6 mm Hg OS. The anterior ocular segment was unremarkable under a slit lamp. The cornea and lens were clear with no signs of inflammation in the anterior chamber. The pupils were equal in diameter and were reactive to light. Relative afferent pupillary defects were not observed. Dilated fundus examination revealed no edema, exudation, or other fundus abnormalities in the retina or the optic papilla (Fig. 1).

**Figure 2.**
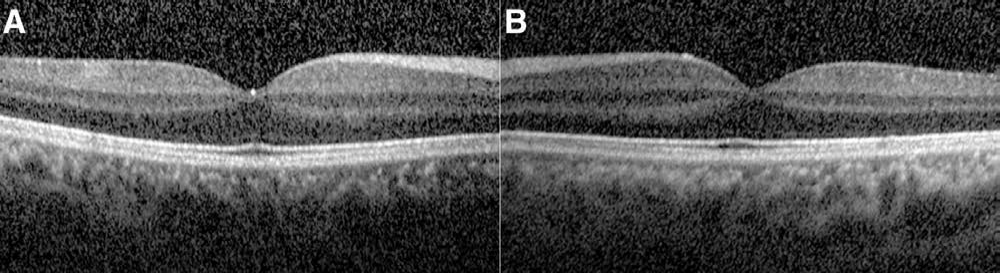
SD-OCT scans of both eyes [(A) the right eye, OD; (B) the left eye, OS]. No obvious changes of macula morphology were found on both eyes under SD-OCT scanning. The thickness of fovea was within normal range. SD-OCT = spectral-domain optical coherence tomography.

**Figure 3.**
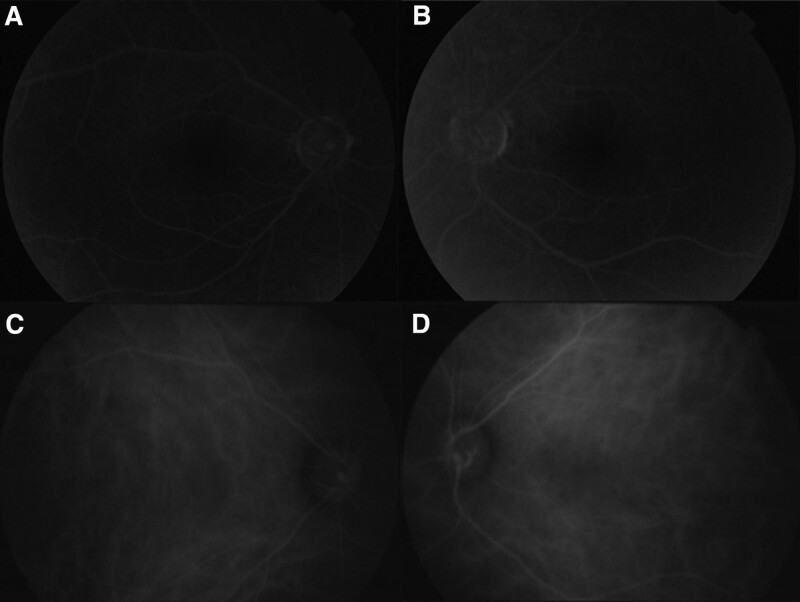
FFA and ICGA of both eyes [(A) the right eye, OD of FFA; (B) the left eye, OS of FFA; (C) the right eye, OD of ICGA; (D) the left eye, OS of ICGA]. No leakage of fluorescence under FFA was found in both eyes throughout all the phases. No abnormality was found under ICGA. FFA = fluorescein angiogram, ICGA = indocyanine green angiography.

No obvious morphological changes were found on B-ultrasound scanning in either eye (data not shown). Optical coherence tomography (OCT, Spectralis HRA + OCT, Heidelberg Engineering, Germany) revealed no detachment, schisis, or hole in the macular retina. The foveal thickness was 259 and 252 μm in the OD and OS groups, respectively (Fig. 2). The thickness of peripapillary retinal nerve fiber layer in both eyes were within normal limits under OCT scanning (data not shown). No leakage of fluorescence was found on fundus fluorescein angiography (FFA) throughout all phases of both eyes. Furthermore, indocyanine green angiography (ICGA) did not reveal any abnormalities in either eye (Fig. 3).

**Figure 4.**
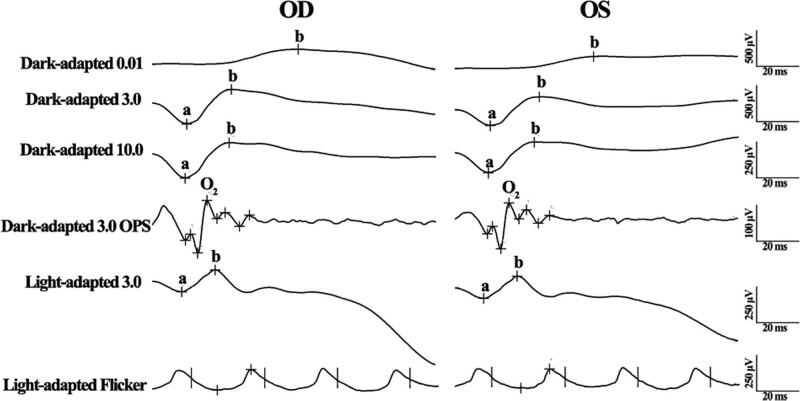
Full-field ERG of both eyes (OD: the right eye; OS: the left eye, OS). The parameters were comparable for both eyes, which were within normative range in general. ERG = electroretinogram.

ERG was performed using a Ganzfeld stimulator (Roland, Germany) according to the International Society for Clinical Electrophysiology of Vision (ISCEV) standard after 30 minutes of dark adaptation with dilated pupils. ERG data revealed no obvious changes in parameters, such as amplitude and peak time, in both dark-adapted and light-adapted responses from both eyes. In addition, the data were comparable between both eyes (Fig. 4).

**Figure 5.**
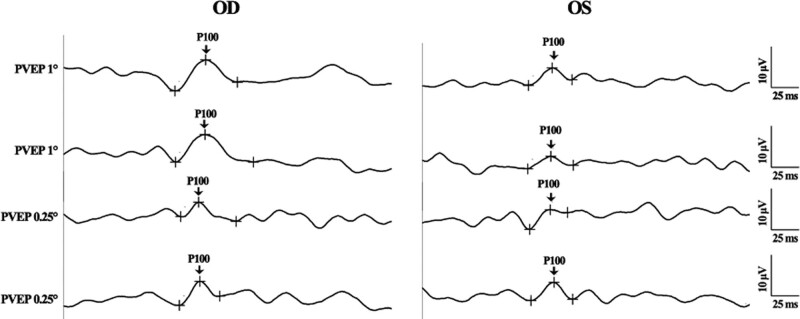
PVEP of both eyes (OD: the right eye; OS: the left eye, OS). A reduced amplitude and a delayed peak time of P100-wave were found in both eyes. The delayed phase of P100-wave was more obvious in the right eye, while the reduced amplitude of P100-wave was more obvious in the left eye. PVEP = pattern visual-evoked potentials.

**Figure 6.**
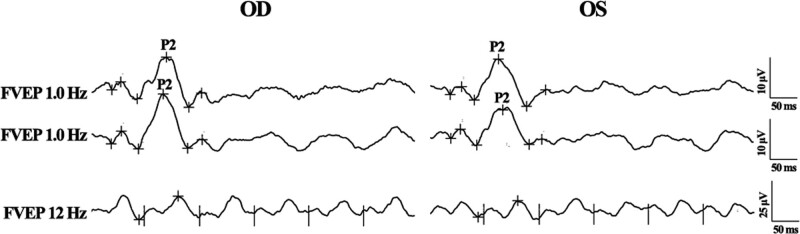
FVEP of both eyes (OD: the right eye; OS: the left eye, OS). The amplitude and peak time of P2 component of FVEP were comparable between the right eye and the left eye, which were both within normative range. FVEP = flash visual-evoked potentials.

The PVEP was recorded according to the ISCEV standard. Reproducibility and reliability were obtained by performing the recording twice. PVEP detected a slightly reduced amplitude and a slightly delayed peak time in the P100-wave of both the 1° (1°) and 0.25° PVEP in both eyes. The delayed peak time of the P100-wave was more obvious in the right eye, whereas the reduced amplitude of the P100-wave was more obvious in the left eye (Fig. 5). The FVEP was performed according to the ISCEV standard with twice recording. Reproducible and reliable waveforms of FVEP were observed in both eyes. The amplitude and peak time of the P2-wave were comparable in both eyes and were within the normative ranges (Fig. 6).

**Figure 7.**
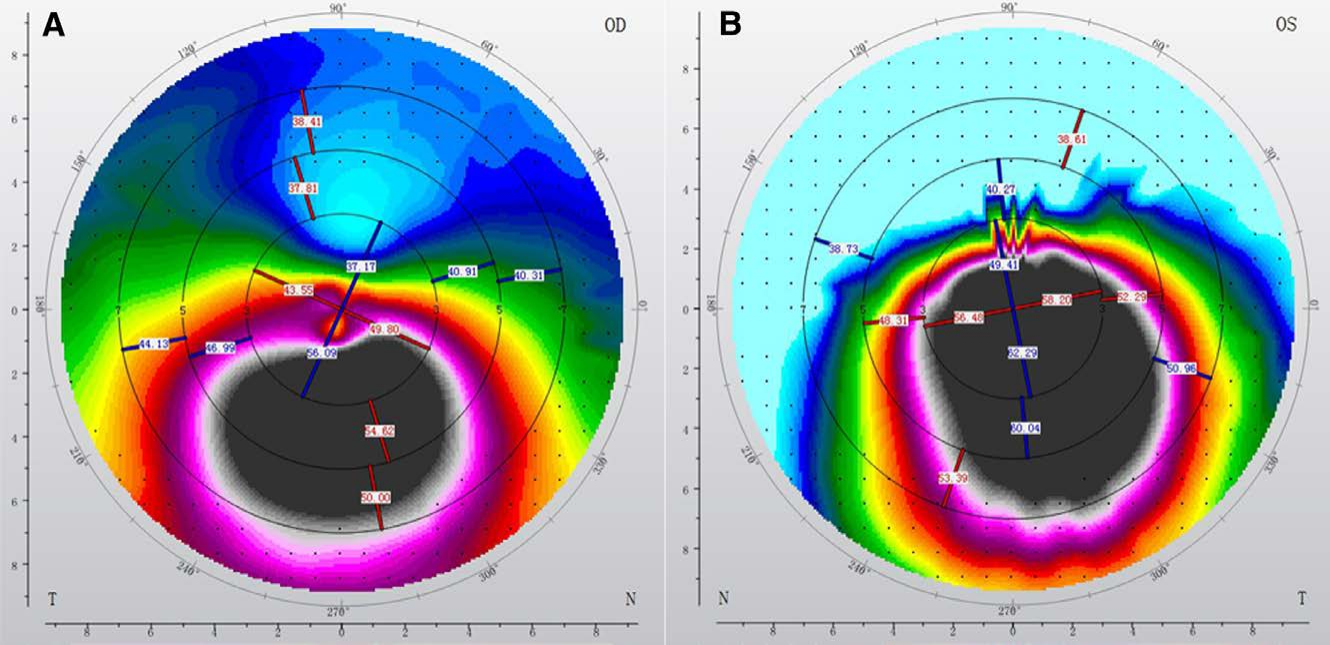
Corneal topography maps [(A) the right eye, OD; (B) the left eye, OS]. Typical bowtie pattern were revealed under corneal topography in both eyes, showing an inferior steep thinning cornea, characteristic of KCN. KCN = keratoconus.

Corneal topography was finally performed, and an asymmetrical bowtie pattern of the cornea was found in both eyes, which was more obvious in the OS (Fig. 7). The patient was finally diagnosed with bilateral KCN and was immediately referred to our superior hospital for transepithelial corneal collagen cross-linking treatment. The BCVA of both eyes remained stable after treatment at follow-up.

## 3. Discussion

In the initial stages, KCN may be asymptomatic. Gradually, its progression causes visual morbidity due to high astigmatism and anisometropia. Patients with KCN usually complain of decreased VA, which cannot be improved by correcting for refractive error.^[11,12]^ The diagnosis of KCN in the initial stage relies on specific methods such as corneal topography.^[13–15]^ In particular, high astigmatism or an asymmetrical bowtie pattern detected in corneal topography is clinically suggestive of KCN.^[16,17]^ As in our case, corneal topography revealed asymmetrical bowtie patterns in the left eye. The refractive error of high myopia and astigmatism in the left eye led to unimproved VA. The left eye was definitively diagnosed as having KCN. Refractive error in the right eye was significantly lower. BCVA of the right eye was 20/20, even with asymmetrical bowtie patterns. The KCN of the right eye might be in the early stage, and the central point of the cornea could retain an intact curvature after correcting for refractive error.^[18]^ The anisometropia between the right and left eyes in our case was in accordance with the asymmetrical bilateral progression of KCN.^[19]^

Decreased vision, which cannot be improved by correcting refractive error, is usually associated with ocular lesions in the posterior segment.^[20]^ As the KCN lesion lies in the cornea, no pathologic changes are found in the posterior segment unless other systemic diseases are present.^[21]^ KCN was not brought about as the reason for the unimproved VA in our case, as we did not find obvious changes in the cornea or lens under slit lamp for the first time. However, we did not obtain any positive clues from any fundus examination. Commonly, electrophysiological examinations are helpful in the diagnosis of unknown reasons for decreased VA.^[9,10]^ ERG is the mass potential of retinal electrical activity that evaluates global retinal function. The influence of ocular media would not be impressive, as the stimuli for ERG are strong flashlights.^[22]^ The ERG results of our case did not reveal any obvious abnormalities in either response, indicating intact retinal function. The OCT scanning with the FFA and ICGA results excluded the possibility of macular lesions, although the multifocus ERG (mfERG) was not performed to provide the electrophysiological activity for the macula.^[23]^

PVEP obtains a waveform with pattern-reversal checkboard stimuli. The P100-wave from PVEP is usually prominent peaks that show relatively little variation between subjects and minimal interocular differences.^[8]^ Abnormalities in the P100-wave parameter, particularly the peak time, are usually associated with optic nerve diseases when patients experience unimproved VA.^[24,25]^ A slightly reduced amplitude and delayed peak time of the P100-wave in both eyes in our case. The ocular lesion was first assumed to lie in the optic nerve or visual pathway. The literature showed that the reduced PVEP amplitude might be caused by light scatter from irregular astigmatism, as light scatter had an effect on the mfERG amplitudes.^[26]^ The peak time of the P100-wave could also be affected by nonpathophysiologic factors such as refractive error, opaque ocular media, and poor fixation.^[8]^ In addition, Geng et al^[27]^ reported that PVEP is susceptible to the influence of visual attention. Thus, PVEP alone could not confirm whether the lesion was in the ocular media or on the posterior segment.

FVEP, which is more variable than PVEP among subjects, usually remains similar between both eyes of an individual subject. FVEP could help assess visual function in patients with ocular media opacities, which would prevent the valid use of PVEP. Usually, the most consistent and robust component in FVEP is the P2-wave.^[8]^ In our case, the peak time and amplitude of the P2-wave in both the eyes were comparable. In addition, these parameters were all within normal ranges.^[28]^ The abnormal PVEP parameters and normal FVEP parameters from our case led us to focus the lesion on the ocular media. In addition, we excluded the possibility of malingering as a reason for the unimproved VA. Furthermore, the ocular media of the patient, including the anterior chamber, lens, and vitreous body, were generally clear. Considering all above situations, the ocular lesion for the unimproved VA in our case was assumed to be existed on the cornea The KCN was brought about as the possible diagnosis, which was finally confirmed by the corneal topography results.

KCN may be accompanied by other ocular disorders, such macular dysfunction, retinitis pigmentosa or Leber congenital amaurosis.^[21,29,30]^ Electrophysiological examinations are useful in evaluating potential diseases under such circumstances. Moschos et al^[31]^ reported that the retinal response density in the mfERG differed significantly between some KCN patients and normal controls, implying the coexistence of impaired macular function. They also applied ERG and VEP in a series of 233 patients with KCN and revealed the presence of diffuse tapetoretinal degeneration in many cases.^[32]^ Nguyen et al^[33]^ diagnosed a 35-year-old male of KCN associated with the Congenital Stationary Night Blindness Type 1, whose ERG recording revealed no response in the dark adaption and a “negative” waveform response. In such circumstances, the low VA of these KCN might not only be attributed to corneal abnormalities but also to defective retinal dysfunction. Preoperative electrophysiological studies of such KCN cases could also provide valuable prognostic information and are crucial to avoid unnecessary treatment, such as corneal transplantation. Fogla et al. discovered an unimproved BCVA after penetrating keratoplasty in the left eye in a bilateral KCN case, as the coexistence of cone-rod retinal dystrophy was found in postoperative ERG results.^[34]^ As no significant dysfunction of the retina and the visual pathway from the ERG and FVEP was found in our case, the patient was predicted to have a good VA after successful treatment of the cornea.

## 4. Conclusion

In summary, we presented the diagnostic process of a KCN case after electrophysiological examinations in combination with other morphological studies. The availability of electrophysiology could help gain a deeper understanding of the clinical picture and prognostic outcome of treatment for KCN. In addition, KCN should be suspected in patients with significant irregular stigmatism when no obvious lesions exist in other ocular parts.

## Correction

When originally published, Weiming Yan appeared incorrectly as the corresponding author. This has been corrected to Meizhu Chen with the email address jumychen@126.com. The rest of the corresponding author location information remains the same.
